# Mirror therapy combined with neuromuscular electrical stimulation for poststroke lower extremity motor function recovery: a systematic review and meta-analysis

**DOI:** 10.1038/s41598-023-47272-9

**Published:** 2023-11-16

**Authors:** Zhen-Han Oh, Chia-Hung Liu, Chih-Wei Hsu, Tsan-Hon Liou, Reuben Escorpizo, Hung-Chou Chen

**Affiliations:** 1 Department of Family Medicine, Shuang Ho Hospital, Taipei Medical University, New Taipei City, Taiwan; 2 Department of Family Medicine, School of Medicine, College of Medicine, Taipei Medical University, Taipei, Taiwan; 3 Department of Physical Medicine and Rehabilitation, Shuang Ho Hospital, Taipei Medical University, No. 291 Zhongzheng Road, Zhonghe District 235, New Taipei City, Taiwan; 4 Department of Physical Medicine and Rehabilitation, School of Medicine, College of Medicine, Taipei Medical University, Taipei, Taiwan; 5 Department of Rehabilitation and Movement Science, College of Nursing and Health Sciences, University of Vermont, Burlington, VT USA; 6 Swiss Paraplegic Research, Nottwil, Switzerland; 7 Center for Evidence-Based Health Care, Shuang Ho Hospital, Taipei Medical University, New Taipei City, Taiwan

**Keywords:** Stroke, Skeletal muscle

## Abstract

The combination of mirror therapy (MT) and neuromuscular electrical stimulation (NMES) has been devised as an intervention method in stroke rehabilitation; however, few studies have investigated its efficacy in lower extremity motor function recovery. In this systematic review and meta-analysis, we examined the effectiveness of combined MT and NMES therapy in improving poststroke walking speed, spasticity, balance and other gait parameters. Randomized controlled trials (RCTs) were selected from PubMed, Cochrane Library, EMBASE, and Scopus databases. In total, six RCTs which involving 181 participants were included. Our findings indicate that MT combined with NMES elicits greater improvement relative to control group in walking speed (SMD = 0.67, 95% confidence interval [CI] 0.26–1.07, P = 0.001), Berg Balance Scale (SMD = 0.72; 95% CI 0.31–1.13; P = 0.0007), cadence (SMD = 0.59, 95% CI 0.02–1.16, P = 0.04), step length (SMD = 0.94, 95% CI 0.35–1.53, P = 0.002), and stride length (SMD = 0.95, 95% CI 0.36–1.54, P = 0.002) but not in modified Ashworth scale (SMD =  − 0.40, 95% CI − 1.05 to 0.26, P = 0.23). Our findings suggest that MT combined with NMES may be a suitable supplemental intervention to conventional therapy in stroke survivors.

## Introduction

Stroke is a cerebrovascular disease. Each year, approximately 9.6 million and 4.1 million cases of ischemic and hemorrhagic strokes, respectively, occur worldwide^1^. Stroke is the second leading cause of death worldwide. Stroke mortality rates have declined due to medical advancements^2^. The number of stroke survivors is higher now than before. From 1990 to 2016, the global age-standardized mortality rate sharply decreased by 36.2%, whereas the global age-standardized incidence decreased by a lower rate of 8.1%; these findings indicate that the burden of stroke is likely to remain high^3^. A substantial proportion of stroke survivors have poststroke impairments, such as movement disorder, sensory impairment, visual defects, and other sequelae that affect independent function^4^. Furthermore, lower extremity motor function, which is commonly impaired after stroke, affects gait and postural performance^5^. Kim et al. reported that the prevalence of lower limb weakness reached up to 72% in stroke survivors^6^. Several rehabilitation approaches exist that can improve lower extremity motor function in stroke survivors, such as dual task exercise, training using functional electrical stimulation, mirror therapy (MT), mental imagery, virtual reality, and robotic interactive therapy^7–12^.

MT is a relatively new therapeutic intervention that is used in clinical rehabilitation for stroke survivors. MT is a form of mental practice and cognitive intervention that excites the primary motor cortex and evokes movement of the affected limb by moving the unaffected limb and receiving mirrored feedback from the motion^13,14^. MT can activate mirror neurons and produce a strong effect on the motor network by increased cognitive penetration in action control^15^. MT facilitates lower extremity motor function recovery, thus improving the motor function and gait perfomance of acute stroke survivors^16,17^. Ji et al. compared an experimental group that underwent MT combined with comprehensive rehabilitation therapy with a control group that underwent sham MT combined with comprehensive rehabilitation therapy, and their results indicated that the experimental group achieved significant post-training gains for their single stance, step length, and stride length performance^16,17^.

Neuromuscular electrical stimulation (NMES) is among the most commonly used interventions in clinical settings for stroke survivors^18,19^. NMES stimulates lower motor neurons by applying electrical stimulation to activate affected muscles and induce muscle contraction. Consequently, NMES may prevent muscle atrophy, maintain muscle tolerance, increase muscle strength, and retrain functional movements^20^. NMES has previously been reported to be effective in improving lower extremity motor function, muscle strength, range of motion, and gait ability among stroke survivors^21–23^.

Both the interventions have shortcomings. Stroke survivors with MT have difficulty in performing spontaneous muscle contractions on their affected limbs. However, NMES can activate affected limbs by stimulating lower motor neurons and subsequently inducing spontaneous muscle contractions^20^. Besides, NMES may reduce the effect of motor relearning due to the mechanism of simple passive repetitive stimulation^24^. Thus, for stroke survivors, NMES combined with voluntary and active training has been suggested to overcome the shortcomings of NMES. Knutson et al. demonstrated that gait training combined with either contralaterally controlled neuromuscular electrical stimulation or cyclic neuromuscular electrical stimulation reduced lower extremity impairment^25^. Moreover, the combination of MT and NMES can overcome the shortcomings of the individual therapies and enhance the effectiveness of stroke rehabilitation by inducing a patient’s voluntary and active participation. Several studies have suggested that MT and NMES exert a positive synergistic effect on the functional recovery of patients with stroke^26,27^.

Although several meta-analyses and systematic reviews have demonstrated the synergistic effects of combination MT and NMES therapy on upper extremity motor function recovery in stroke survivors^28,29^, few studies have investigated the effects of combination MT and NMES therapy on lower extremity motor function recovery. Thus, in our study, we investigated the effectiveness of combination MT and NMES therapy in lower extremity motor function recovery in stroke survivors.

## Methods

### Study design and registration

This systematic review and meta-analysis was conducted in accordance with Preferred Reporting Items for Systematic Reviews and Meta-analysis (PRISMA) guidelines^30^. The PRISMA checklist is presented in Supplementary Appendix A. The protocol was prospectively registered in the international prospective register of systematic reviews (PROSPERO) under registration number CRD42022370696.

### Eligibility criteria

We included randomized controlled trials (RCTs) that applied the combination of MT and NMES therapy to patients with stroke that assessed the outcomes of lower extremity motor function and impairment. We excluded RCTs that used isolation treatment or evaluated the outcomes of upper extremities or diseases other than stroke. RCTs that were protocols, conference papers, or animal studies were also excluded. No language-related restrictions were applied during article selection.

### Data sources and retrieval

RCTs were identified by two reviewers independently on the basis of the title, abstract, and full text of the studies. The electronic databases used included PubMed, Cochrane Library, EMBASE, Scopus, and Google Scholar. The following keywords for the disease and intervention in combination were used: (mirror OR MT) AND (electric* OR electro OR current) AND (leg OR foot OR (lower AND (extremit* OR Limb*)) OR walk*” OR gait) AND (stroke OR cerebrovascular OR CVA OR brain vascular OR ICH OR ((infarct* or hemorrhag*) and (brain or cerebral OR cerebell* OR pons OR pontine OR medulla* OR MCA OR PCA OR ACA)). The databases were searched from their date of inception until October 26, 2022.

### Data items and data extraction

The following parameters were extracted from each RCT by two reviewers independently for the MT + NMES and control groups: number of patients, sex, phase of stroke, follow-up period, baseline therapy for both intervention and control groups, placement and duration of treatment, and assessed outcome measures. The MT + NMES group comprised stroke survivors who underwent MT + NMES therapy and conventional therapy, whereas the control group comprised stroke survivors who underwent conventional therapy with or without sham therapy (sham MT, sham NMES, or sham MT with sham NMES). Our meta-analysis included RCTs involving stroke survivors who had experienced hemiplegia at any stage of stroke and exhibited various degrees of impairment in the motor function of lower extremities. These impairments include problems related to ambulation, balance, *muscle* tone, and spasticity of lower extremity. Chronic stroke phase and subacute stroke phase referred to the disease duration of more than 6 months and the disease duration of 3–6 months from stroke occurrence, respectively. All outcome measures assessed immediately after treatment completion were analyzed in our study. Outcomes that were included in two or more RCTs were assessed in our meta-analysis. The primary outcome was walking speed. The secondary outcomes assessed in the present study were Berg Balance Scale (BBS) score, modified Ashworth scale (MAS) score, and several gait parameters (i.e., cadence, step length, and stride length).

### Risk-of-bias assessment

The methodological quality of each study was examined using the Physiotherapy Evidence Database (PEDro) scale, a valid and widely used measurement tool to evaluate risk of bias^31^. In total, 11 items were included in the PEDro scale for each RCT: (1) eligibility criteria and source of participants, (2) random allocation, (3) concealed allocation, (4) baseline comparability, (5) blinding of participants, (6) blinding of therapists, (7) blinding of assessors, (8) adequate follow-up (more than 85%), (9) intention-to-treat analysis, (10) between-group statistical comparisons, and (11) reporting of point measures and measures of variability. Each item is rated yes or no (which correspond to 1 and 0 points) determined by whether an item is clearly met by a study. However, eligibility criteria and source of participants were excluded from the calculation due to external validity. Therefore, a PEDro score rating from 0 to 10 was obtained by adding up the ratings of the other 10 items. The risk-of-bias assessment was conducted by two independent authors (Z.H. Oh and C.H. Liu), and the quality of each study was classified as poor (score of 0–3), fair (score of 4 or 5), good (score of 6–8), or excellent (score of 9 or 10)^32^.

### Quality of evidence

The methodological quality of evidence was assessed using the Grading of Recommendations, Assessment, Development, and Evaluation (GRADE), a valid and widely used measurement tool^33^. The certainty of the included RCTs was determined on the basis of their study design, risk of bias, inconsistency, imprecision, indirectness, publication bias, and effect sizes along with their trends. Two independent authors (Z.H. Oh and C.H. Liu) screened studies, extracted data, and examined the quality of evidence. The GRADE framework categorizes the quality of evidence into four levels: very low, low, moderate, and high.

### Statistical analysis

Data management and analysis were performed using Review Manager (RevMan) software (version 5.4.1, the Cochrane Collaboration, London, UK). The study was performed in accordance with PRISMA guidelines^30^. All relevant data with varying scales were converted to a single scale and are expressed as the standard mean difference (SMD). To account for variations in the number of stroke survivors, the phase of stroke experienced by survivors, and the duration of follow-up among the RCTs included in the analysis, data were pooled using a random-effects model for all comparisons. The precision of the effect sizes was reported as 95% confidence intervals (CIs). Heterogeneity between studies was investigated using the I^2^ statistic, with I^2^ > 25%, 50%, and 75% indicating low, moderate, and high heterogeneity, respectively^34^. The effects of high heterogeneity were analyzed in sensitivity analysis to determine their significance. Results were considered statistically significant at *P* ≤ 0.05 in the *z*-tests of equivalence.

## Results

### Study selection

**Figure 1:**
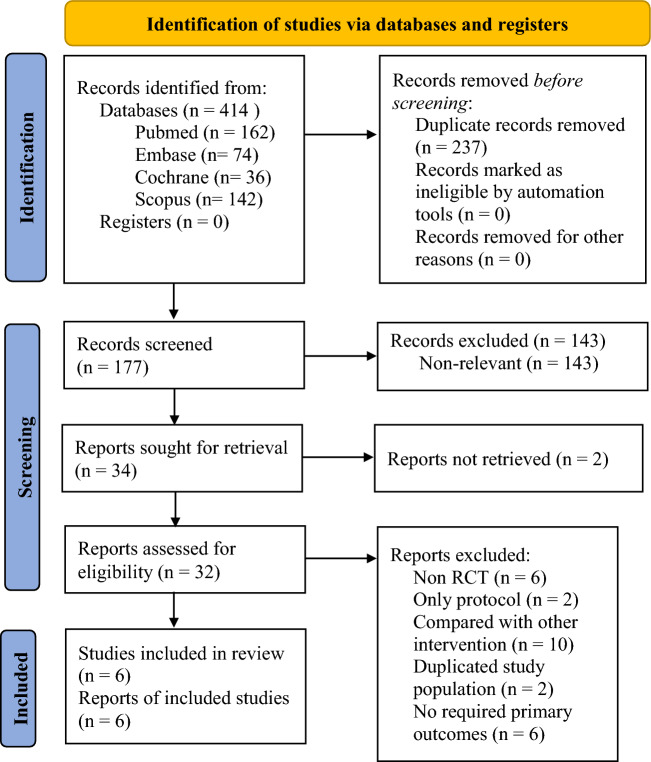
PRISMA flow diagram of study selection process. n, number.

After application of the aforementioned search terms, a total of 414 articles were initially retrieved. Of these studies, 237 were excluded as duplicates and 143 articles were excluded after title and abstract screening. The remaining 34 RCTs were screened, of which 28 were excluded for various reasons: reports not retrieved, nonrandomized study design, only protocol, compared with other intervention, duplicated study population, no required primary outcomes. Finally, five parallel studies^35–39^ and one crossover study^40^ that investigated the effectiveness of combination MT and NMES therapy on lower extremity motor function recovery in stroke survivors were included in the meta-analysis. A detailed flowchart of the study selection process is presented in Fig. 1^30^.

### Study characteristics

The six selected studies were published between 2014 and 2021^35–40^. A total of 181 patients (91 patients in the MT + NMES group and 90 in the control group) were included. All patients in both the groups received conventional therapy as the baseline treatment. Four studies investigated the chronic phase of stroke^35,36,38,39^, one study investigated the subacute phase of stroke^40^, and the remaining study investigated the acute and chronic phases of stroke^37^. Three studies used sham therapy and conventional therapy in the control group^35,37,38^, and the other three studies used only conventional therapy in the control group^36,39,40^. All studies used a parallel-group study design except the study by Salhab^40^, which used a crossover study design. All studies reported the effectiveness of MT + NMES in improving the walking speed of patients with stroke. Three trials assessed balance by using the Berg Balance Scale (BBS)^36,38,39^. Three studies used the modified Ashworth scale (MAS), which is a performance-based scale used to assess the level of impairment and motor function in patients with stroke^36–38^. Gait parameters, including cadence, step length, and stride length, was assessed in two studies^35,38^. The included RCTs did not report any adverse effects. The main characteristics of these six RCTs are summarized in Table 1.

**Table 1:** Characteristics of the included studies.

Study	Stroke phase	Follow-up period	Study design	Treatment protocol and setting	Sham therapy in control group	Assessed outcome measure in this meta-analysis
Kim et al., 2021^39^	Chronic	At the end of intervention (after 8 weeks)	Parallel	**Experimental group, MT + NMES (n = 20; M/F : 12/8)** MT: Repeated flexion + extension of the knee and ankle while observing oneself in a mirror NMES: Involving the use of Myomed 134; intensity, 10–20 mA; frequency, 35 Hz 30 min/session**;** 5 sessions/week for 8 weeks **Control group (n = 20; M/F : 10/10)** **Baseline therapy for both intervention and control groups** Conventional therapy: neurodevelopmental physical therapy 30 min/session; 5 sessions/week for 8 weeks	No	10 MWT, BBS
Lee et al., 2019^38^	Chronic	At the end of intervention (after 4 weeks)	Parallel	**Experimental group, MT + NMES (n = 15; M/F : 11/4)** MT: Repeated dorsiflexion of both ankles with a mirror box measuring 50 × 70 cm^2^ NMES: Use Mesh Sock with the P1 program, which comprises 15 min of electric stimulation at a frequency of 100 Hz and a pulse width of 300 μs as well as 15 min of electric stimulation at a frequency of 15 Hz and a pulse width at 300 μs 30 min/session; 5 sessions/week for 4 weeks **Control group (n = 15; M/F : 10/5)** **Baseline therapy for both intervention and control groups** Conventional therapy (gait training) + NMES in MT + NMES group; Conventional therapy (gait training) + sham NMES in control group 30 min/session; 5 sessions/week for 4 weeks	Yes: (sham mirror box without a reflective mirror, sham AES device was set not to operate)	Walking speed, MAS, BBS, cadence, step length, stride length
Xu et al., 2017^37^	Acute & chronic	At the end of intervention (after 4 weeks)	Parallel	**Experimental group, MT + NMES (n = 23; M/F : 7/16)** MT: Repeated flexion + extension of the ankles while observing oneself in a 60 × 90 cm^2^ mirror NMES: Use 5 × 5 cm^2^ electrodes with frequency, 50 Hz; intensity, 10 mA; duration, 5 s 30 min/session; 5 sessions/week for 4 weeks **Control group (n = 23; M/F : 8/15)** **Baseline therapy for both intervention and control groups** Conventional therapy: physical and occupational therapy, neurodevelopment facilitation 4 h/session; 5 sessions/week for 4 weeks	Yes: (use of the non-reflective side of the mirror)	10MWT, MAS
Lee et al., 2016^36^	Chronic	At the end of intervention (after 4 weeks)	Parallel	**Experimental group, MT + NMES (n = 14; M/F : 7/7)** MT: Repeated dorsiflexion of both ankles while observing oneself in a 50 × 70 cm^2^ mirror NMES involving the use of Microstim (Medel GmbH, Germany), which comprises an external switch and surface electrodes (5 × 5 cm^2^); frequency, 35 Hz; pulse duration, 250 μs; duration, 0.2 s 5 sessions/week for 4 weeks **Control group (n = 13; M/F : 7/6)** **Baseline therapy for both intervention and control groups** Conventional therapy: muscle facilitation, balance and gait training, and task-specific functional training 60 min/session; 5 sessions/week for 4 weeks	No	6MWT, MAS, BBS
Salhab et al., 2016^40^	Subacute	At the end of intervention (after 2 weeks)	Cross-over	**Experimental group, MT + NMES (n = 18; M/F : 10/8)** MT: Repeated ankle dorsiflexion while observing oneself in a 70 × 40 cm^2^ mirror NMES: Involve the usage of symmetrical rectangular biphasic waveform current; frequency < 10 Hz; pulse width, 300 μs; total stimulation time, 16 min/session, with 6/6 duty cycle 1st period of intervention with MT + NMES: 40 min/session; 4 sessions/week for 2 weeks Undergo crossover after washout period of 1 week 2nd period of intervention with Conventional therapy: 40 min/session; 4 sessions/week for 2 weeks **Control group (n = 18; M/F : 10/8)** **Baseline therapy for both intervention and control groups** Conventional therapy: stretching, active and passive mobilization 10 min/session; 4 sessions/week for 2 weeks in each period of intervention	No	10MWT
Ji et al., 2014^35^	Chronic	At the end of intervention (after 6 weeks)	Parallel	**Experimental group, MT + NMES (n = 10; M/F : 6/4)** MT: Repeated dorsiflexion for 10 s followed by 5 s rest while observing oneself in a 60 × 90 cm^2^ mirror NMES: Using Microstim with surface electrodes (50 × 50 mm^2^), a stimulator, and a foot switch 20 min/session; 5 sessions/week for 6 weeks **Control group (n = 10; M/F : 6/4)** **Baseline therapy for both intervention and control groups** Conventional therapy: PNF neurodevelopment technique 30 min/session; 5 sessions/week for 6 weeks	Yes: (without application of NMES, mirror covered with white cloth)	Walking speed, cadence, step length, stride length

6MWT, 6-m walking test; 10MWT, 10-m walking test; BBS, Berg Balance Scale; F, female; M, male; MT, mirror therapy; MAS, modified Ashworth scale; n, number of participants; NMES, neuromuscular electrical stimulation; PNF, proprioceptive neuromuscular facilitation.

### Risk-of-bias assessment

The PEDro scale was used to assess the risk of bias and evaluate the quality of the selected RCTs. This assessment was conducted by two authors by independently. All studies were classified as having good quality and obtained scores of 6–8 points for overall quality. All studies reported random allocation, adequate baseline comparability, adequate follow-up, between-group statistical comparisons, point estimates, and variability measures for at least one key outcome. Only one study applied concealed allocation. Therapists and participants were not blinded in any study. However, assessors were blinded in three studies. In five studies, intention-to-treat analysis was conducted. Detailed results are illustrated in Table 2.

**Table 2:** Summary of methodological quality based on PEDro scale.

	1	2	3	4	5	6	7	8	9	10	11	Total	Rating
Kim et al., 2021^39^	V	V		V				V	V	V	V	6	Good
Lee et al., 2019^38^	V	V		V			V	V	V	V	V	7	Good
Xu et al., 2017^37^	V	V	V	V			V	V	V	V	V	8	Good
Lee et al., 2016^36^	V	V		V			V	V		V	V	6	Good
Salhab et al., 2016^40^	V	V		V				V	V	V	V	6	Good
Ji et al., 2014^35^		V		V				V	V	V	V	6	Good

Items: 1, eligibility criteria and source of participants; 2, random allocation; 3, concealed allocation; 4, baseline comparability; 5, blinded participants; 6, blinded therapists; 7, blinded assessors; 8, adequate follow-up; 9, intention-to-treat analysis; 10, between-group comparisons; 11, point estimates and variability. *Not included in the calculation of the total score. Methodological quality: excellent, 9–10 points; good, 6–8 points; fair, 4–5 points; poor, 0–3 points; yes (V), 1 point; no (), 0 points.

### Quality of evidence

The quality of evidence was evaluated using the GRADE system. This assessment was also conducted independently by two authors. According to the GRADE methodology, the evidence quality was determined to be moderate for the assessment of walking speed. However, for the secondary outcomes, the GRADE assessment indicated a low quality of evidence for BBS and gait parameters, whereas it indicated a very low quality of evidence for MAS. Detailed results are illustrated in Table 3.

**Table 3:** Summary of methodological quality of evidence according to GRADE.

Certainty assessment							Number of patients		Effect		Certainty	Importance
Number of studies	Study design	Risk of bias	Inconsistency	Indirectness	Imprecision	Other considerations	MT + NMES	Control	Relative (95% CI)	Absolute (95% CI)		
Walking speed												
6	Randomized trials	Serious^a^	Not serious	Not serious	Not serious	None	100	99	–	SMD **0.67 higher** (0.26 higher to 1.07 higher)	⨁⨁⨁◯ Moderate	IMPORTANT
Berg balance scale												
3	Randomized trials	Serious^a^	Not serious	Not serious	Serious^b^	None	49	48	–	SMD **0.72 higher** (0.31 higher to 1.13 higher)	⨁⨁◯◯ Low	IMPORTANT
Modified ashworth scale												
3	Randomized trials	Serious^a^	Serious^c^	Not serious	Serious^b^	None	52	51	–	SMD **0.4 lower** (1.05 lower to 0.26 higher)	⨁◯◯◯ Very low	IMPORTANT
Gait parameter: cadence												
2	Randomized trials	Serious^a^	Not serious	Not serious	Serious^b^	None	25	25	–	SMD **0.59 higher** (0.02 higher to 1.16 higher)	⨁⨁◯◯ Low	IMPORTANT
Gait parameter: step length												
2	Randomized trials	Serious^a^	Not serious	Not serious	Serious^b^	None	25	25	–	SMD **0.94 higher** (0.35 higher to 1.53 higher)	⨁⨁◯◯ Low	IMPORTANT
Gait parameter: stride length												
2	Randomized trials	Serious^a^	Not serious	Not serious	Serious^b^	None	25	25	–	SMD **0.95 higher** (0.36 higher to 1.54 higher)	⨁⨁◯◯ Low	IMPORTANT

CI: confidence interval; SMD: standardized mean difference.

^a^All studies that did not involve participant and therapist blinding because of specific treatment characteristics.

^b^All studies with a small sample size and a wide confidence interval.

^c^The heterogeneity is high, and *I*^2^ = 63%.

### Synthesis of results

#### Walking speed

**Figure 2:**
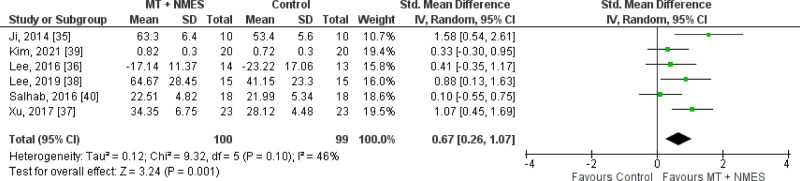
Walking speed. MT, mirror therapy; NMES, neuromuscular electrical stimulation; 95% CI, 95% confidence interval.

Walking speed was assessed in all the studies^35–40^. Our results revealed that the MT + NMES group exhibited significantly higher levels of improvement than did the control group (SMD = 0.67, 95% CI 0.26–1.07, n = 199, *I*^2^ = 46%). A forest plot of walking speed is presented in Fig. 2.

#### Berg balance scale

**Figure 3:**
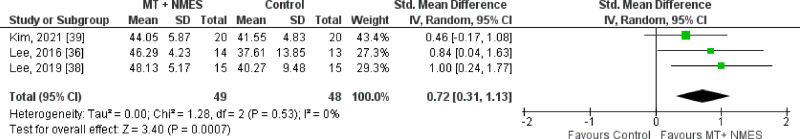
Berg balance scale. MT, mirror therapy; NMES, neuromuscular electrical stimulation; 95% CI, 95% confidence interval.

Three studies assessed balance by using the BBS^36,38,39^. These studies involved 49 patients in the MT + NMES group and 48 patients in the control group. Our meta-analysis of BBS indicated that improvement in the MT + NMES group was significantly higher than that in the control group (SMD = 0.72; 95% CI 0.31–1.13; n = 97; I^2^ = 0%). A forest plot for BBS is presented in Fig. 3.

#### Modified Ashworth scale

**Figure 4:**
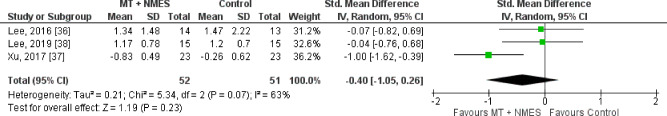
Modified Ashworth scale. MT, mirror therapy; NMES, neuromuscular electrical stimulation; 95% CI, 95% confidence interval.

Assessments of spasticity using the MAS were reported in three studies^36–38^. These studies involved 52 patients in the MT + NMES group and 51 patients in the control group. No significant difference between the MT + NMES and control groups was observed (SMD =  − 0.40, 95% CI − 1.05 to 0.26, n = 103, *I*^2^ = 63%). A forest plot for spasticity assessed using MAS is shown in Fig. 4.

#### Gait parameters

**Figure 5:**
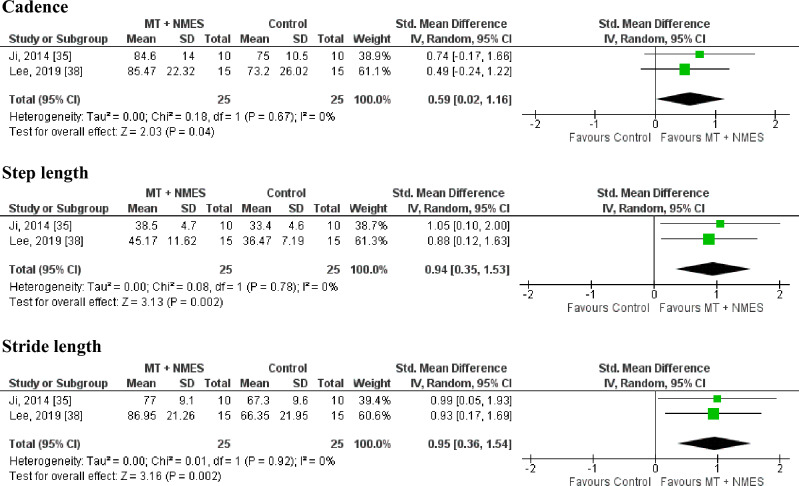
Gait parameters. MT, mirror therapy; NMES, neuromuscular electrical stimulation; 95% CI, 95% confidence interval.

Gait parameters (cadence, step length, and stride length) was assessed in two studies^35,38^. These studies involved 25 patients in the MT + NMES group and 25 patients in the control group. The MT + NMES groups exhibited significantly higher levels of improvement than did the control groups for cadence, step length, and stride length (SMD = 0.59, 95% CI 0.02–1.66, n = 50, *I*^2^ = 0%; SMD = 0.94, 95% CI 0.35–1.53, n = 50, *I*^2^ = 0%; SMD = 0.95, 95% CI 0.36–1.54, n = 50, *I*^2^ = 0%, respectively). A forest plot for gait parameters is presented in Fig. 5.

## Discussion

In this systematic review and meta-analysis, we analyzed the effectiveness of the combination of MT and NMES therapy on improving poststroke lower extremity motor function and impairment. Six outcomes were assessed in these studies: walking speed, BBS, MAS, cadence, step length, and stride length. These are reliable and widely used measures of gait performance and balance among patients with stroke. Aside from the MAS scores, our findings indicate that MT + NMES is more effective than control group in improving lower extremity motor function. Information on adverse events was not reported in any of the studies. Therefore, the combination of MT with NMES therapy can be considered a safe and well-tolerated intervention.

Mirror visual feedback, as a core mechanism of MT, can lead to higher excitability of the primary motor cortex that might be helpful in the rehabilitation of patients with stroke^13^. To our knowledge, excitement of the ipsilateral primary motor cortex that projects to the affected limb may be induced by the movement of the unaffected limb with visual feedback using MT^41^. By contrast, NMES therapy can enhance the activation of the sensory motor cortex in patients with stroke and may subsequently increase muscle strength and endurance and reduce spasticity^19^. Stroke survivors with MT have difficulty performing spontaneous muscle contractions on affected limbs, and the effects of NMES therapy are limited by its simple passive repetitive stimulation mechanism. In the present study, MT was combined with NMES therapy as an intervention for patients with stroke. This combination can possibly promote a synergetic effect that overcomes the limitations of each individual therapy.

Levels of spasticity, as measured by the MAS, did not significantly improve in either the treatment or control group. Several previous studies have revealed the ineffectiveness of MT alone in improving the muscle tone of upper and lower limbs. Spasticity, which involves a complex pathophysiology, may not be sufficiently controlled or affected by the visual feedback provided by MT^17,42,43^. NMES therapy applied in combination with MT may increase the conductivity of synapses between the pyramidal tract and anterior horn cells and thus reduce spasticity^37^. Although MAS scores in our study did not significantly increase, we observed slightly more positive results in the MT + NMES group than in the control group. These results are possibly due to the relatively small sample size; only 103 stroke survivors were included for the assessment of motor impairment using the MAS. Additional research with a larger sample size may provide more definitive evidence.

The risk of bias in the studies selected in this meta-analysis was assessed using the PEDro scale. Due to the nature of MT and NMES therapy, none of the included RCTs implemented therapist blinding. Furthermore, participant blinding was not implemented in any of the studies. Although three studies reported the usage of sham therapy in the control group^35,37,38^, we do not consider sham MT using nonreflective mirrors^37,38^ or mirrors covered with a white cloth^35^ to be adequate for participant blinding, because patients allocated to sham MT will realize their classification into the control group. Furthermore, only one study implemented concealed allocation; therefore, our meta-analysis had an increased risk of introduced bias. Although the PEDro scores for all RCTs included in this meta-analysis were determined to be of good quality, the aforementioned biases must be considered when interpreting our findings.

The quality of evidence in this meta-analysis was assessed using the GRADE system. As mentioned earlier, none of the included RCTs implemented therapist blinding and participant blinding due to the nature of MT and NMES therapy. Consequently, all the included RCTs received a risk of bias rating of “serious” according to the GRADE system. The quality of evidence was higher for the primary outcome—walking speed, than for the secondary outcomes. This discrepancy was mainly because of the relatively small sample sizes and wide confidence intervals for all the secondary outcomes, which resulted in a “serious” rating for imprecision in the GRADE assessment.

In addition to comparing MT + NMES therapy with conventional therapy, three of the studies also compared MT + NMES therapy with MT alone in terms of improving walking speed^35,37,39^. In these studies, patients in the MT + NMES group demonstrated significantly higher walking speeds than those in the MT only group. Additional studies are required to investigate the effectiveness of combination MT and NMES therapy versus MT alone for improving poststroke lower extremity motor function and impairment. Furthermore, we found that no RCT investigated the effectiveness of combination MT and NMES therapy versus NMES alone in improving poststroke lower extremity motor function and impairment. Additional research is warranted to compare the effectiveness of these two interventions.

The present study has several strengths. First, this is the first meta-analysis to clarify that the combination of MT and NMES therapy significantly improves the walking performance of patients with stroke; this finding can be expanded in future studies. Second, the PEDro scores reported by all included RCTs were determined to be of good quality (scores of 6–8 points). Additionally, according to the GRADE system, our primary outcome, which was walking speed, was assigned a rating of moderate quality of evidence. This suggests high internal validity and reliability of the results obtained from these RCTs, thereby providing substantial scientific evidence to support the findings.

This study has several limitations. First, only six RCTs were included. The strength of the results may be limited by the relatively small sample size. Second, outcomes were assessed immediately after treatment. Whether the findings are applicable over the long term is unclear. Third, blinding of therapists and participants was not implemented in all included studies, potentially resulting in performance bias. Additional larger-scale studies with high methodological quality are required to overcome these limitations.

In conclusion, combination MT and NMES therapy is a promising intervention for the improvement of poststroke lower extremity motor function, and it may be a suitable supplemental intervention to conventional therapy for stroke survivors. However, because the small sample size of this meta-analysis could have reduced the overall evidence level, additional studies on this topic are warranted. Furthermore, to clarify the synergetic effects of the combination of MT and NMES therapy, more RCTs should be conducted to compare the effectiveness of the combination of MT and NMES therapy versus NMES or MT alone in improving the lower extremity motor function and impairment of stroke survivors.

### Supplementary Information

Supplementary Information.

## Data Availability

As this is a systematic review and meta-analysis, all extracted data are already available in published manuscripts. All generated data are included in the article.
